# Case studies in physiology: Impact of a long‐distance hike on the Pacific Crest Trail on arterial function and body composition in a highly fit young male

**DOI:** 10.14814/phy2.14767

**Published:** 2021-03-04

**Authors:** Thomas C. Heinbockel, Daniel H. Craighead

**Affiliations:** ^1^ Department of Integrative Physiology The University of Colorado Boulder Boulder CO USA

**Keywords:** body composition, endothelial function, endurance exercise

## Abstract

The Pacific Crest Trail (PCT) is a 4265‐km hiking trail that extends from the US‐Mexican border to the US‐Canadian border through the mountain ranges of western North America. Individuals who hike the entire length of the trail in one season (4–6 months) perform long daily exercise durations while exposed to extreme environmental temperatures, high altitudes, intense solar radiation, and the consumption of calorie‐rich, nutrient‐poor diets. This case study reports changes in arterial function and body composition in a subject before and after a 112‐day long‐distance hike of the PCT. Brachial artery flow‐mediated dilation, a measure of vascular endothelial function, decreased from: 6.97% to 5.00%. Carotid‐femoral pulse wave velocity, a measure of aortic stiffness, increased from 5.39 to 5.76 m/s. Dual‐energy x‐ray absorptiometry scans detected no major changes in total‐body bone mineral density, fat mass, or lean mass, although there were minor, unfavorable changes in some subregions of the body. It is important for individuals completing a long‐distance hike to be aware of the potential deleterious changes associated with large volumes of exercise and consuming a high‐calorie, low‐quality diet.

## Graphical abstract

The subject trekked 4,052km over 112 days. Tests were performed before and after the trek.

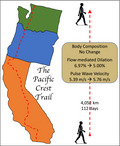

## 1 INTRODUCTION

The Pacific Crest Trail (PCT) is a continuous, 4265‐km mountainous footpath stretching from the United States (US)‐Mexican border through California, Oregon, and Washington to the US‐Canadian border. The main mountain ranges of the PCT include the Sierra Nevada of the US state of California and the Cascade Range of the states of California, Oregon, and Washington. The trail ascends and descends a total of about 250,000 m with an impressive range from 43 m at The Bridge of the Gods in Cascade Locks, Oregon, to 4009 m at the summit of Forester Pass in California. Of the millions of people who set foot on the PCT each year, only a few hundred attempt and complete a thru‐hike—a single season, continuous hike of the entire length. Thus, completion of the PCT represents an impressive physical feat. This noteworthy accomplishment requires a thru‐hiker to perform long daily exercise durations in extreme environmental temperatures, high altitudes, and intense solar radiation while simultaneously traversing rugged wilderness and carrying a heavy backpack filled with all essential gear and food.

A successful thru‐hike also requires increased caloric intake well above basal metabolic rate. Hikers routinely hitchhike into towns at road crossings every 4–5 days to gather food and supplies, repair gear, and recover. Arriving into town with significant reductions in body weight, food resupply is the main priority during town‐stops with the goal of consuming as many calories as possible. For this subject, in‐town calories largely come from foods such as cheeseburgers, pizza, ice cream, and other fast food items. Quintessential food for this hiker to carry on‐trail are lightweight yet calorically dense, to minimize pack weight, such as candy bars, pre‐packaged pastries, chips, nuts, and jerky. While these foods are high in calories, they contain few essential nutrients.

Most hikers travel in a northbound direction from Mexico to Canada and require the majority of the 6‐month snow‐free season between April and September (Pacific Crest Trail Association, 2019). The subject in this report was a highly fit, 25‐year‐old male who completed a 112‐day hike of the PCT from May 11, 2019 to August 30, 2019, completing the PCT ~30% faster than the average hiker. With a narrowed time‐window to complete the trail, this hiker needed to be very fit to maintain high daily exercise volumes. The subject also needed to consume a high number of calories (~5500 kcal/day) to accommodate the large volume of exercise. The purpose of this case study in physiology was to document how the combination of high volumes of physical activity and consuming a nutrient‐poor, calorically‐dense diet over a prolonged period of time alters important health markers, particularly arterial function, body composition, and bone mineral density.

## 2 MATERIALS AND METHODS

All laboratory testing took place at the Integrative Physiology of Aging Laboratory and the Clinical Translational Research Center at the University of Colorado Boulder. The subject provided informed consent as part of a larger, ongoing IRB‐approved study. Baseline measurements were taken 8 days prior to beginning the thru‐hike and post‐testing measurements were taken 7 days after completing the thru‐hike. Cardiovascular measures were performed hydrated, fasted, and in the mornings after abstaining from caffeine and vigorous exercise. Systolic blood pressure (SBP) and diastolic blood pressure (DBP) were measured using an automated device (Cardiocap/5, GE).

Endothelial function was assessed with brachial artery flow‐mediated dilation (FMD_BA_), as described previously (Early et al., 2017; Philips IE33 Ultrasound System). An ultrasound probe (Philips L9‐3 Linear Array Probe) was placed on the upper arm of the subject. After a 1‐min, baseline measure of brachial artery diameter was obtained; a blood pressure cuff that was placed on the forearm was inflated to occlude blood flow to the hand for 5 min. Brachial artery diameter was measured for 30 s prior to release of the cuff and 2 min following cuff release. Ultrasound images were recorded at end‐diastole using an ECG‐triggered image capture system (Vascular Imager; Medical Imaging Applications LLC) and analyzed offline using automated edge‐detection and wall‐tracking software (Brachial Analyzer; Medical Imaging Applications LLC). FMD was measured as the absolute (mm) and percent‐change in brachial artery diameter from baseline to peak diameter on the ultrasound machine.

Carotid‐femoral pulse wave velocity (CFPWV), a measure of aortic stiffness, was assessed using applanation tonometry (Townsend et al., 2015). With tonometers (Millar SPT‐301 Non‐invasive Pulse Tonometer) placed at the carotid artery and the femoral artery, pulse wave velocity was calculated as the distance between the two probes divided by the time delay of the pulse wave between the two locations. Resting heart rate (HR) was derived from the tonometry measurements. Body composition (bone mineral density [BMD], fat mass and lean mass) was measured by dual‐energy x‐ray absorptiometry (DEXA). Maximal oxygen consumption (VO_2_max) was assessed on a treadmill and performed at an altitude of 1655 m using the Balke protocol (Evans et al., 1995).

**FIGURE 1:**
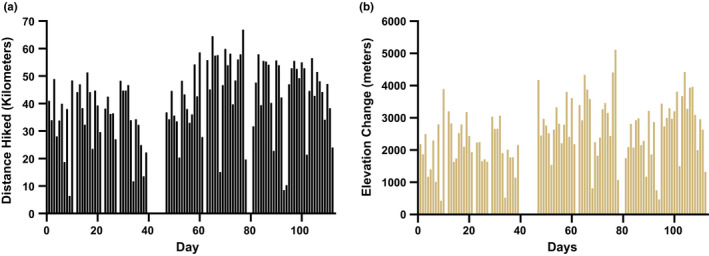
(a) Distance hiked per day (km) and (b) change in elevation hiked per day (m) by the subject while completing the Pacific Crest Trail

The subject hiked a total of 4052 km in 112 days. A total of 212 k were skipped in California and Oregon due to trail closures and health reasons when the subject developed foot pain too severe to continue without significant risk of long‐term injury. When this occurred, the subject hitchhiked into town at the nearest road crossing, spent ~1 day in town recovering, and then resumed hiking at another road crossing further along the trail. During this 112‐day period, 92 days were spent hiking and 20 days were spent off‐trail as recovery days. Daily hiking distance and elevation change are shown in Figure 1. A summary of the physiological stimulus of the 112‐day hike is given in Table 1.

**TABLE 1:** Exercise stimulus

	Per hiking day	Total	1‐day maximum
Distance hiked (km)	44.1	4052	66.9
Elevation gain and loss (m)	2730	251,203	5115
Hiking duration (min)	486.6	44,766	750
Minutes with HR above 80% HR_max_	28.1	2585	119
Estimated caloric expenditure during activity (kcal)	3315	304,981	5970
Steps	54,715	5,033,789	81,557
Push‐ups	151	13,884	1000

The subject collected all field measurements using a Garmin Fenix 5X multisport GPS watch with built‐in wrist‐based heart rate monitoring and the GutHook App for the iPhone, which provided official distances along the route. Data were recorded daily and stored on the subject's mobile device.

## 3 RESULTS

Subject characteristics are given in Table 2. The subject was young, highly fit, and had a low body fat percentage.

**TABLE 2:** Subject characteristics

	Pre‐PCT	Post‐PCT
Age	25	—
Sex	Male	—
Height (cm)	184	—
Weight (kg)	80.1	79.3
BMI (kg/m^2^)	23.7	23.4
Body fat percentage (%)	5.8	6.0
VO_2_max (ml/kg/min)	61.3	—

### 3.1 Cardiovascular function

Resting diameter of the brachial artery was 4.50 mm pre‐PCT and 4.62 mm post‐PCT. FMD_BA_ was 0.31 mm/6.97 ∆% pre‐PCT and 0.23 mm/5.00 ∆% post‐PCT. Peak diameter of the brachial artery was 4.81 mm pre‐PCT and 4.85 mm post‐PCT. There was an increase in CFPWV from 5.39 to 5.76 m/s pre‐ to post‐PCT, possibly indicating a modest increase in aortic stiffness. There was no change in resting SBP (115 mmHg pre‐PCT, 116 mmHg post‐PCT) or DBP (66 mmHg pre‐PCT, 67 mmHg post‐PCT) and all blood pressures were within the normotensive range. Resting HR was 60 beats per minute pre‐PCT and 68 beats per minute post‐PCT.

### 3.2 Body composition

Detailed body composition data are presented in Table S1. The hiker experienced small changes in body weight (−0.99%), BMD (−0.56%), and body fat percentage (+3.45%) pre‐ to post‐PCT. Although there was no significant change in whole‐body BMD, there were two subregions of the body that exhibited changes after the 112 days of hiking. The BMD of the spine decreased by 5.17%, and the BMD of the pelvis decreased by 3.76% pre‐ to post‐PCT. Total‐body BMD T‐Score was 0.5 pre‐PCT and 0.4 post‐PCT, and total‐body BMD Z‐Score was 0.4 pre‐PCT and 0.3 post‐PCT, making these changes not substantial to total‐body BMD. The total fat mass of the hiker increased by 1.79% from baseline to post‐PCT. In the legs and arms, there was a decrease in fat mass by 6.3% and 3.8%. Alternatively, there was a 9.0% increase in the fat mass of the trunk from pre‐ to post‐PCT. The hiker experienced a decrease in total body lean mass by 1.2% after the PCT, coming mostly from a 6.2% decrease in the arms and a 4.5% decrease in the legs.

Additionally, the subject performed daily push‐ups to maintain upper body strength, completing 282 push‐ups per hiking day during the first 56 days. On the 57th day, 1000 push‐ups were performed, after which, shoulder pain developed. For the following 13 days, daily push‐ups were reduced to 66 per day. After this, all push‐ups were discontinued in an attempt to preserve all energy.

## 4 DISCUSSION

In this case study, we evaluated the effects of a long‐distance hike on the PCT on arterial function and body composition in a highly‐fit, 25‐year‐old male. Our primary finding was that FMD_BA_, a marker of endothelial function, decreased. Additionally, there were multiple minor changes in CFPWV, BMD, fat mass, and lean mass. In addition to the extreme exercise load, the subject performed to complete the PCT, the individual consumed a very poor diet for the duration of the hike. The results of this case study suggest that the combination of extreme exercise and poor diet may lead to negative changes in health markers.

### 4.1 Endothelial function

The decrease in FMD_BA_ from 6.97∆% to 5.00∆% indicates a likely decrease in endothelial function. As FMD_BA_ is a risk factor for cardiovascular diseases (Yoshida et al., 2006), this may indicate heightened disease risk following the thru‐hike. Decreased FMD_BA_ may not have been expected because of the well‐established benefits of chronic endurance exercise training on endothelial function. Exercise training that is similar in mode and intensity, but not duration, to the hiking exercise stimulus in this case study is sufficient to significantly improve endothelial function (Early et al., 2017), which is aerobic‐based with a total weekly volume >150 min/week, including high intensities, and performed in healthy/fit individuals under 30 years of age for periods greater than 12 weeks. However, others have shown that extreme exercise volumes may lead to impaired endothelial function (Durand & Gutterman, 2014). It is important to note that the resting brachial diameter of our participant increased while completing the PCT. Increased arterial diameter is a known effect of aerobic exercise (Green et al., 2012) and may in part contribute to the decrease in FMD_BA_ expressed as a percent. However, it should be noted that absolute dilation of the brachial artery decreased in our participant as well. We did not measure shear stress in this study, thus was cannot rule out that the observed reduction in FMD_BA_ was a result of decreased shear stress and not a direct change in endothelial function per se.

Although results from human trials on the effects of high‐fat/high‐sugar diets on endothelial function are variable, a Western‐style diet can impair endothelium‐dependent dilation in mice (Donato et al., 2012). The diet consumed by the hiker was similar to a standard Western‐style diet and could be responsible for the observed decreased FMD_BA_. Importantly, we cannot uncouple the contribution of high volumes of exercise and poor diet in this case study, so it may be that the combination of these two stimuli decreases FMD_BA_, even in young, fit individuals.

### 4.2 Aortic stiffness

A modest increase in CFPWV from 5.39 to 5.76 m/s was observed. Resting BP, a factor that can modulate aortic stiffness (Cohen et al., 2020; Tan et al., 2018), increased by 1 mmHg in our subject, supporting that the increase in aortic stiffness could potentially be due to structural modifications. Our subject did exhibit an 8 beat per minute increase in resting HR. Increased resting HR has been shown to be related to increased CFPWV; however, this is only in adults with higher CFPWV than our subject (Papaioannou et al., 2019). While there is a controversy around the contributions of HR to CFPWV, the majority of evidence indicates that the relation is primarily mediated by the influence of HR on BP, which again only increased by 1 mmHg in our subject. Therefore, it is unlikely, although possible, that the increase in resting HR was the primary mediator of the increased CFPWV observed in our subject. However, we appreciate that the observed change in CFPWV (0.37 m/s) is only marginally larger than our measurement variability (0.30 m/s) and understand that this increase in aortic stiffness may be negligible.

Endurance training tends to decrease arterial stiffness (Sardeli et al., 2018) in both young (Madhura & Sandhya, 2012) and older (Jablonski et al., 2015) adults. As this hiking was a form of aerobic endurance exercise training, we could have expected to see beneficial effects on aortic stiffness. However, extreme volumes of physical activity (Vlachopoulos et al., 2010) and unhealthy diets (García‐Hermoso et al., 2018) have been shown to increase aortic stiffness. Thus, the combination of these two factors likely led to this modest increase in aortic stiffness. Importantly, this subject exhibited a healthy CFPWV at baseline, making additional exercise‐induced improvements in aortic stiffness unlikely (Oudegeest‐Sander et al., 2013). Therefore, more research in the area is warranted.

### 4.3 Body composition

#### 4.3.1 Bone mineral density

Although the overall decrease in total‐body BMD was minor, spine and pelvis decreases were non‐negligible. However, even with these decreases in regional BMD, the subject's total‐body BMD remained within the normal range (*T*‐ and *Z*‐scores >−1; NIH Osteoporosis and Related Bone Diseases National Resource Center^2018^) post‐PCT. Although the subject's BMD was normal post‐PCT, the rate of decline was likely faster than would be observed over the same time period of normal lifestyle in a young adult male (Hanschin & Stern, 1995) and is potentially concerning. Considering that the subject was carrying a 9–21 kg backpack while hiking, it is surprising that this load‐bearing exercise did not maintain/increase total‐body, spine, and pelvis BMD, as is often observed with load‐bearing exercise (Snow et al., 2001). However, repetitive and prolonged exercise (i.e., long‐distance hiking) may not improve BMD due to desensitization of the bone cells to the stimulus (Turner & Robling, 2003). In addition, unhealthy dietary patterns are associated with low BMD (Denova‐Gutiérrez et al., 2018). The decreases in BMD observed in subregions in this subject may be explained by the unhealthy dietary pattern and an exercise load that was not completely protective to prevent this decrease. Had the load‐bearing exercise stimulus not been present, BMD may have decreased even further.

#### 4.3.2 Fat mass

A body fat percentage of 4%–6% in males is considered the physiological minimum before negative physiological changes occur (Friedl et al., 1994). With 5.8% body fat at baseline, the hiker prioritized a high caloric intake (~5500 kcal/day) to help prevent a negative caloric balance. To accommodate this increase in caloric intake during the hike, the hiker had to make changes to their diet. Before the hike, the hiker was mainly consuming rice, fresh vegetables, chicken, and beef. During the hike, the hiker consumed more calorically dense food items such as candy, pastries, nuts, jerky, dried fruit, ramen noodles, instant mashed potatoes, protein bars, and peanut butter. As was desired, there was no change in total‐body fat mass. However, fat mass was redistributed from the arms and legs to the trunk. The use of trekking poles by this hiker allowed the arms to share the workload of locomotion with the legs (Saunders et al., 2008). Thus, the arms were also subjected to extreme exercise loads. In a previous study, 20 weeks of cycling exercise decreased subcutaneous fat in the legs more than in the arms, suggesting that fat mass can decrease preferentially in active muscles (Wilmore et al., 1999). With both arms and legs participating in all hiking locomotion, it was expected that fat mass would be reduced in the arms and legs. This redistribution of fat mass in this subject allowed the limbs to become more economical, due to their smaller mass (Myers & Steudel, 1985), while maintaining a high enough total‐body fat mass to prevent negative physiological changes.

#### 4.3.3 Lean mass

The hiker experienced a negligible decrease in total‐body lean mass of 1.2% from pre‐ to post‐PCT. Interestingly, leg lean mass decreased by 4.5% and arm lean mass decreased by 6.2%. Although not as powerful a stimulus as resistance training, chronic aerobic endurance training (i.e. long‐distance hiking) can induce muscle hypertrophy (Grgic et al., 2019). Even though the subject spent ~28 min/day above 80% of maximum heart rate, and a 9–21‐kg pack was worn at all times, this was an insufficient stimulus to maintain lean mass from pre‐PCT exercise training and was actually a degree of detraining. The subject performed 282 push‐ups per hiking day during the first 56 days in an attempt to attenuate the loss of upper‐body muscle mass that is common amongst long‐distance hikers. These push‐ups likely had a protective effect on arm lean mass (Kohiruimaki et al., 2019). Had no push‐ups been performed, it is possible that the loss of arm lean mass would have been more severe. We appreciate that the push‐up strategy employed by this hiker may have been more aggressive than necessary to prevent loss of upper body muscle mass. It is possible that a more conservative approach of less push‐ups, performed every other day and while wearing a backpack, could be sufficient to preserve muscle mass without causing injury and termination of push‐ups altogether.

## 5 LIMITATIONS AND FUTURE DIRECTIONS

This study was limited by including only a single subject; however, the speed at which this subject completed the PCT allowed a unique look at the physiological effects of large volumes of exercise and a poor‐quality, high‐calorie diet. We cannot uncouple the contributions of these two significant stimuli, and we do not have a detailed dietary record from the subject. FMD_BA_ and CFPWV were only measured once at each timepoint; therefore, changes may partially be due to the day‐to‐day variability of these measurements. Additionally, we did not measure shear rate to compliment the FMD measurements. Subsequent studies investigating the effects of long‐distance hiking should measure changes in traditional cardiovascular disease risk factors (e.g., blood pressure). Additional testing sessions during and after the hike would allow for investigation of the time course of changes in arterial function and body composition.

## 6 CONCLUSIONS

During a 112‐day hike along the PCT, extreme daily volumes of physical activity coupled with a poor diet resulted in a reduction in FMD_BA_, an increase in CFPWV, and minor unfavorable changes to body composition. Our findings suggest that large volumes of exercise do not compensate for a poor diet and/or may provide diminishing returns and prove detrimental to physiological function. These findings have implications for others attempting long‐distance thru‐hikes or similar tests of endurance. Importantly, individuals with poor arterial function or low bone mineral density should take extra precautions when engaging in long‐distance hiking in order to maintain proper physiological function.

## CONFLICT OF INTEREST

The authors declare no competing interests.

## AUTHOR CONTRIBUTION

T.C.H. and D.H.C. conceived and designed research; T.C.H. and D.H.C. collected data; T.C.H. and D.H.C. analyzed data; T.C.H. wrote manuscript; T.C.H. and D.H.C. edited and revised manuscript and approved final version of manuscript.
